# Hantavirus Disease: A Comprehensive Narrative Review of Epidemiology, Pathogenesis, Diagnosis, and Treatment

**DOI:** 10.7759/cureus.111618

**Published:** 2026-06-27

**Authors:** Anies Ahmed, Mariyam Indawala, Suraiya Khan, Barun Kumar, Sameer Chauhan, Murali M. R., Vimal Satodiya, Rahul V. C. Tiwari, Heena Tiwari

**Affiliations:** 1 Department of Oral Medicine and Radiology, Himachal Institute of Dental Sciences, Paonta Sahib, IND; 2 Department of Prosthodontics, Rutgers School of Dental Medicine, Houston, USA; 3 Department of Public Health, Johns Hopkins Bloomberg School of Public Health, Baltimore, USA; 4 Department of Oral and Maxillofacial Surgery, Bharati Vidyapeeth (Deemed to be University) Dental College and Hospital, Navi Mumbai, IND; 5 Department of Prosthodontics, K. M. Shah Dental College and Hospital, Sumandeep Vidyapeeth (Deemed to be University), Vadodara, IND; 6 Department of Psychiatry, JSS Academy of Higher Education and Research, Mysuru, IND; 7 Department of Psychiatry, Inland Psychiatric Medical Group, Lancaster, USA; 8 Department of Oral and Maxillofacial Surgery, Narsinhbhai Patel Dental College and Hospital, Sankalchand Patel University, Visnagar, IND; 9 Department of Blood Cell, Commisionerate of Health and Family Welfare, Government of Telangana, Hyderabad, IND

**Keywords:** disease management, epidemiology, hantavirus cardiopulmonary syndrome, hantavirus infections, hemorrhagic fever with renal syndrome

## Abstract

Hantavirus infection is a rodent-borne zoonotic disease of considerable public health importance. The disease is associated with a wide spectrum of clinical manifestations and continues to attract increasing attention because of its impact on human health and its evolving epidemiological patterns. This narrative review summarizes current knowledge regarding the epidemiology, virology, pathogenesis, clinical features, diagnosis, management, and prevention of hantavirus infection. A comprehensive literature search was conducted using major electronic databases, including PubMed/MEDLINE, Scopus, Embase, Web of Science, and Google Scholar. Relevant peer-reviewed articles, reviews, clinical studies, surveillance reports, and guidelines were evaluated and synthesized to provide an updated overview of the subject. This narrative review discusses the interactions among environmental factors, rodent reservoirs, and human exposure that influence disease occurrence and transmission. Key aspects of disease pathogenesis, clinical presentation, diagnostic approaches, therapeutic considerations, and preventive measures are summarized. Current management strategies remain largely supportive, while ongoing research continues to explore novel diagnostic tools, therapeutic options, and preventive interventions. In conclusion, hantavirus infection remains an important public health concern. Continued efforts toward improved surveillance, timely diagnosis, enhanced research, and effective preventive strategies are essential for better disease control and improved patient outcomes.

## Introduction and background

Hantavirus infection is an emerging zoonotic disease of global public health importance caused by enveloped RNA viruses belonging to the genus* Orthohantavirus *of the family *Hantaviridae *[1]. These viruses are primarily transmitted to humans through the inhalation of aerosolized excreta, saliva, or urine of infected rodents, which serve as natural reservoirs [2]. Since the first recognized outbreaks, hantavirus infections have attracted considerable scientific attention because of their high morbidity and mortality rates, diverse clinical manifestations, and potential for rapid progression to severe cardiopulmonary or renal complications [3,4]. The disease mainly manifests as two major clinical syndromes: hemorrhagic fever with renal syndrome (HFRS), predominantly reported in Europe and Asia, and hantavirus cardiopulmonary syndrome (HCPS), mainly observed in the Americas [5,6]. Both syndromes are characterized by increased vascular permeability and intense immune-mediated responses, although their clinical severity and organ involvement may vary according to the viral strain.

The epidemiology of hantavirus infection has evolved significantly over recent decades because of ecological disturbances, climate change, urbanization, deforestation, and increasing human encroachment into rodent habitats [3]. These environmental and occupational factors have contributed to the emergence and re-emergence of hantavirus outbreaks in several regions across the world. Furthermore, changing rodent population dynamics and improved surveillance systems have enhanced the recognition of previously underdiagnosed infections [7]. Despite advances in virology and molecular diagnostics, hantavirus infection remains a diagnostic challenge because of its nonspecific early symptoms, which often mimic other febrile illnesses, such as dengue, leptospirosis, influenza, and COVID-19 [8].

The pathogenesis of hantavirus infection is complex and primarily involves endothelial dysfunction, dysregulated immune activation, and cytokine-mediated capillary leakage [9]. Early diagnosis and prompt supportive management are crucial because no universally approved specific antiviral therapy is currently available. Preventive strategies mainly focus on rodent control, environmental sanitation, and public awareness of exposure risks [10]. In this context, the present narrative review aims to comprehensively summarize the current perspectives on the epidemiology, pathogenesis, clinical manifestations, diagnostic approaches, and management strategies of hantavirus infection, while also highlighting recent advances and future directions in research and disease control of hantavirus infection.

## Review

Search strategy and data sources

This narrative review was conducted to comprehensively summarize the current evidence regarding the epidemiology, pathogenesis, diagnosis, and management of hantavirus infections. A structured literature search was performed using multiple electronic databases, including PubMed/MEDLINE, Scopus, Web of Science, Google Scholar, and Embase. Relevant articles published from January 2000 to March 2026 were considered for inclusion to ensure coverage of both foundational and recent hantavirus research developments.

The search strategy employed combinations of Medical Subject Headings (MeSH) terms and free-text keywords related to hantavirus infection. The principal search terms included “Hantavirus,” “Hantavirus infection,” “Hemorrhagic fever with renal syndrome,” “Hantavirus cardiopulmonary syndrome,” “HCPS,” “HFRS,” “epidemiology,” “pathogenesis,” “diagnosis,” “clinical manifestations,” “treatment,” “management,” “antiviral therapy,” “rodent-borne diseases,” and “zoonotic viral infections.” Boolean operators, such as AND and OR, were used to refine and combine the search terms appropriately. Manual screening of reference lists from eligible articles and relevant review papers was performed to identify further pertinent studies.

Inclusion and exclusion criteria

Articles were selected based on their relevance to the review objectives. Original research articles, systematic reviews, meta-analyses, clinical studies, case series, surveillance reports, and authoritative guidelines focusing on hantavirus infection were included. Studies discussing epidemiological trends, molecular mechanisms, immunopathogenesis, clinical presentation, laboratory diagnosis, imaging findings, therapeutic approaches, and preventive strategies were considered.

Articles published in languages other than English, conference abstracts without full-text availability, duplicate publications, editorials lacking substantial scientific data, and studies unrelated to human hantavirus infection were excluded from the review. Preference was given to peer-reviewed and high-quality publications with significant scientific contributions.

Study selection and data extraction

Titles and abstracts retrieved from the database search were screened for their relevance. Full-text articles deemed potentially eligible were reviewed in detail. Relevant information regarding study characteristics, geographical distribution, viral strains, transmission patterns, pathogenesis, diagnostic modalities, treatment strategies, and clinical outcomes was extracted and organized thematically.

Data synthesis

The collected literature was narratively synthesized to provide a comprehensive overview of the current understanding of hantavirus infections. The findings were categorized into major domains, including epidemiology, virology, pathogenesis, clinical manifestations, diagnostic approaches, management strategies, and preventive measures. Recent advances and emerging perspectives in hantavirus research are also highlighted to provide updated insights into this evolving infectious disease.

Epidemiology of hantavirus infection

Hantavirus infection is an important rodent-borne zoonotic disease with widespread global distribution and significant regional variability in incidence, clinical severity, and circulating viral strains [3]. The epidemiology of hantavirus infection is strongly influenced by ecological, climatic, occupational, and socioenvironmental factors. Rodents serve as the primary natural reservoirs, and transmission to humans mainly occurs through the inhalation of aerosolized virus particles from infected rodent urine, feces, or saliva. Human infection is generally accidental and closely associated with environmental exposure to rodent habitats [11]. According to Watson et al. [11], hantavirus infections represent an increasing public health concern because of expanding rodent populations, changing environmental conditions, and increased human encroachment into natural ecosystems. The global distribution of hantavirus species reflects the geographical distribution of their rodent hosts, resulting in distinct regional epidemiological patterns.

Asia accounts for the majority of reported HFRS cases worldwide, with China contributing nearly 70%-90% of the global burden. Hantaan and Seoul viruses are the principal etiological agents in China and Korea. Large-scale epidemiological surveillance studies in China have demonstrated that HFRS remains endemic in several provinces, particularly in the northeastern and central regions [5,7]. Tian and Stenseth [12] emphasized the major role of ecological and climatic factors such as temperature variation, rainfall, vegetation cover, rodent density, and land-use changes in influencing hantavirus transmission dynamics in China. Seasonal peaks are often associated with fluctuations in rodent populations and increased agricultural activity. Bai et al. [13], in a systematic review of ecological studies from mainland China, further demonstrated a significant association between ambient temperature and HFRS incidence, although the strength of the association varied according to geographical scale and study methodology.

In East Asia, Korea continues to report endemic cases of HFRS, predominantly caused by the Hantaan virus, particularly among rural populations, agricultural workers, and military personnel exposed to rodent-infested environments. Seoul virus infections have also been identified in urban settings because of the widespread distribution of infected Rattus norvegicus populations. In Japan, hantavirus infections are relatively infrequent and occur sporadically, with a substantially lower disease burden than in other East Asian countries, such as China and Korea. These regional epidemiological differences are largely influenced by rodent host distribution, ecological conditions, and human exposure patterns [7,14].

Several European countries have reported increasing hantavirus activity in recent decades. The predominant hantavirus species in Northern and Central Europe is the Puumala virus, which is mainly associated with bank voles (*Myodes glareolus*). Finland, Sweden, Germany, Belgium, Slovenia, and the Balkan countries frequently report outbreaks of nephropathia epidemica, a milder form of HFRS caused by the Puumala virus [15]. Olsson et al. [16] highlighted the heterogeneous geographical distribution of hantavirus reservoir hosts across Europe and emphasized that environmental conditions greatly influence regional disease occurrence. Climatic factors such as mild winters, increased seed production, and changes in forest ecosystems contribute to rodent population expansion and subsequent outbreaks. In Germany and France, periodic increases in hantavirus incidence have been associated with mast years characterized by increased beech seed production, which enhances rodent survival and reproduction [17].

In Russia and Eastern Europe, the Dobrava-Belgrade and Puumala viruses are important causes of HFRS. Dobrava virus infections are generally associated with severe disease manifestations and high mortality rates. Outbreaks have also been documented in Balkan countries, including Slovenia, Croatia, Serbia, Bosnia and Herzegovina, and Greece [18].

In the Americas, hantavirus cardiopulmonary syndrome was first recognized in 1993 during an outbreak in the Four Corners region of the United States of America. The outbreak was linked to the Sin Nombre virus carried by deer mice (*Peromyscus maniculatus*). Since then, numerous cases have been reported in North and South America [6]. The United States and Canada continue to report sporadic HCPS cases, mainly in rural and semi-rural regions with high rodent exposure. Occupational exposure, camping, farming, and cleaning rodent-infested buildings are recognized risk factors [19].

South America has reported several highly pathogenic hantavirus strains, including the Andes, Laguna Negra, and Araraquara viruses. Argentina and Chile have experienced repeated outbreaks of Andes virus-associated HCPS, characterized by high mortality rates and occasional human-to-human transmission [20]. Brazil has also reported increasing numbers of hantavirus infections, particularly in agricultural regions undergoing rapid environmental transformation and deforestation. Studies from South America have shown that ecological disturbances, urban expansion, and agricultural intensification contribute significantly to altered rodent-human interactions and increased transmission risk [7].

Environmental and ecological determinants play a central role in the epidemiology of hantavirus. Zeier et al. [21] proposed a paradigm shift in the understanding of hantavirus ecology by emphasizing the dynamic interactions between rodent hosts, environmental variability, and human behavioral factors. Climate change has emerged as a major contributor to the emergence and re-emergence of hantaviruses. Increased rainfall, flooding, rising temperatures, and altered vegetation patterns influence rodent breeding and migration, as well as viral transmission dynamics. Natural disasters and ecological disturbances may increase human exposure to infected rodents. Occupational exposure is a significant global risk factor. Farmers, forestry workers, military personnel, laboratory workers, and individuals involved in outdoor activities are particularly vulnerable to this disease. Poor housing conditions, inadequate sanitation, food contamination, and overcrowded living environments further contribute to the transmission risk, especially in low-resource settings [22].

Although human-to-human transmission is generally uncommon, Andes virus infection in Argentina has demonstrated limited person-to-person spread, particularly among close household contacts and healthcare workers [23]. This unique epidemiological feature differentiates the Andes virus from most other hantavirus species and highlights the need for vigilant infection control practices during outbreaks. Overall, the epidemiology of hantavirus infection reflects the complex interplay between environmental conditions, rodent ecology, climate variability, and human behavior. Continued surveillance, ecological monitoring, and interdisciplinary research are essential for understanding regional disease patterns and implementing effective prevention and control strategies.

Virology and pathogenesis

Hantaviruses are enveloped, single-stranded, negative-sense RNA viruses that belong to the family *Hantaviridae *and genus *Orthohantavirus *[1]. The viral genome comprises three RNA segments: small (S), medium (M), and large (L). The S segment encodes the nucleocapsid protein, the M segment encodes two envelope glycoproteins (Gn and Gc), and the L segment encodes the viral RNA-dependent RNA polymerase (RdRp). These structural proteins play essential roles in viral attachment, replication, immune evasion, and pathogenesis [24]. Hantaviruses primarily infect endothelial cells, macrophages, dendritic cells, and epithelial cells without causing direct cytopathic destruction, which distinguishes hantavirus infection from many other viral hemorrhagic fevers [5].

Transmission to humans occurs mainly through the inhalation of aerosolized rodent excreta contaminated with infectious viral particles. Following entry into the respiratory tract, the virus initially infects pulmonary endothelial cells and disseminates through the bloodstream. Viral entry into host cells is mediated by interactions between hantaviral glycoproteins and cellular receptors, particularly β3-integrins, β1-integrins, and decay-accelerating factor (DAF/CD55). Pathogenic hantaviruses exhibit a strong affinity for endothelial cells lining the blood vessels, contributing to widespread vascular dysfunction [25].

The central pathogenic mechanism of hantavirus infection is increased vascular permeability resulting from endothelial dysfunction, rather than direct endothelial cell destruction. Viral replication activates both innate and adaptive immune responses, leading to the excessive production of inflammatory mediators and cytokines. Infected endothelial and activated immune cells release pro-inflammatory cytokines, such as tumor necrosis factor-α, interleukin-1, interleukin-6, interferon-γ, and chemokines, which collectively contribute to capillary leakage syndrome. Increased vascular permeability results in plasma extravasation, tissue edema, hypotension, hemoconcentration, and impaired organ perfusion [26,27].

Cell-mediated immunity plays a crucial role in the pathogenesis of diseases. Cytotoxic CD8+ T lymphocytes are activated in response to infected endothelial cells and contribute to endothelial injury through immune-mediated mechanisms. Although this immune response is important for viral clearance, excessive activation may exacerbate tissue damage and increase disease severity. Elevated levels of circulating cytokines and immune complexes are associated with severe clinical manifestations and poor prognosis. Additionally, complement activation and dysregulated coagulation pathways contribute to thrombocytopenia, hemorrhagic manifestations, and microvascular injury [5,6,26,27].

Platelet dysfunction is another characteristic of hantavirus infection. Pathogenic hantaviruses directly interact with platelets and endothelial integrins, resulting in platelet consumption, thrombocytopenia, and impaired vascular integrity. Increased endothelial permeability and platelet abnormalities contribute to the hemorrhagic manifestations observed in severe cases. Studies have also demonstrated that vascular endothelial growth factor (VEGF) and bradykinin pathways may further enhance endothelial leakage during infections [28].

The pathophysiological manifestations vary according to the specific hantavirus strain and the clinical syndrome involved. The kidneys are the principal target organs in hemorrhagic fever with renal syndrome (HFRS). Renal endothelial injury and capillary leakage result in proteinuria, hematuria, oliguria, acute kidney injury (AKI), and electrolyte disturbance. Tubulointerstitial hemorrhage and renal medullary congestion are common pathological findings in AKI. Severe HFRS may progress to hypotensive shock, disseminated intravascular coagulation, and multiple organ dysfunction [5,29].

In hantavirus cardiopulmonary syndrome (HCPS), pulmonary capillary endothelial dysfunction is predominant. Increased pulmonary vascular permeability leads to the rapid accumulation of fluid within the alveolar spaces, resulting in noncardiogenic pulmonary edema, hypoxemia, respiratory failure, and cardiogenic shock. Myocardial depression and circulatory collapse may occur in severe cases. HCPS is often characterized by abrupt clinical deterioration and carries a high mortality rate, despite intensive supportive care [6].

Recent advances in molecular virology have improved our understanding of hantavirus-host interactions and immune dysregulation. Ongoing research is focused on identifying viral virulence factors, host susceptibility markers, and the molecular pathways involved in endothelial injury. These findings may contribute to the future development of targeted antiviral therapies, immunomodulatory strategies, and effective vaccines against hantavirus infections.

Clinical manifestations

The clinical presentation of hantavirus infection varies according to the viral strain and disease syndrome. The incubation period typically ranges from one to six weeks. Early symptoms are generally nonspecific and include fever, chills, headache, myalgia, fatigue, nausea, vomiting, abdominal pain, and dizziness. These prodromal manifestations often resemble those of influenza, dengue fever, leptospirosis, or other viral febrile illnesses, making early diagnosis difficult [30].

Hemorrhagic fever with renal syndrome progresses through five clinical phases: febrile, hypotensive, oliguric, diuretic, and convalescent. Patients may develop petechiae, mucosal bleeding, thrombocytopenia, renal dysfunction, oliguria, and shock due to this condition. Severe cases can progress to acute kidney injury and multi-organ failure [29,31].

Hantavirus cardiopulmonary syndrome generally begins with a prodromal febrile illness, followed by rapid cardiopulmonary deterioration. Patients may develop cough, dyspnea, tachypnea, hypoxemia, pulmonary edema, and circulatory collapse within a short period of time. Despite advances in intensive care management, the mortality rate of HCPS remains high. Laboratory findings commonly include thrombocytopenia, hemoconcentration, leukocytosis, elevated liver enzymes, and abnormal coagulation parameters [32,33].

Diagnostic approaches

Early and accurate diagnosis of hantavirus infection is essential for timely clinical management and improved patient outcomes. Diagnosis is primarily based on clinical suspicion, epidemiological exposure history, laboratory findings, and confirmatory serological and molecular tests.

Serological assays remain the cornerstone of diagnosis in these cases. Enzyme-linked immunosorbent assay (ELISA) for the detection of hantavirus-specific IgM and IgG antibodies is widely used because of its sensitivity and accessibility [34,35]. Immunofluorescence assays and immunoblotting techniques may also assist in the diagnosis. Reverse transcriptase-polymerase chain reaction (RT-PCR) allows for the direct detection of viral RNA during the acute phase and is particularly useful for early diagnosis and viral genotyping [36].

Routine laboratory investigations frequently demonstrate thrombocytopenia, leukocytosis, elevated hematocrit levels, proteinuria, and renal impairment. Imaging studies, such as chest radiography and computed tomography, may reveal bilateral interstitial infiltrates or pulmonary edema in HCPS [32]. Differential diagnoses should include dengue, leptospirosis, malaria, influenza, COVID-19, septicemia, and other causes of acute febrile illness with thrombocytopenia.

Management and preventive strategies

Currently, no universally approved specific antiviral therapy exists for hantavirus infection, and clinical management remains primarily supportive. Early diagnosis, prompt hospitalization, and intensive supportive care are critical determinants of patient survival, particularly in severe cases of HFRS and HCPS. Patients with significant respiratory compromise, hemodynamic instability, or renal dysfunction often require intensive care unit admission for close monitoring and organ support.

Supportive management includes maintaining adequate oxygenation, careful fluid and electrolyte balance, vasopressor therapy for shock, renal replacement therapy in cases of acute kidney injury, and mechanical ventilation for respiratory failure. In HCPS, meticulous fluid management is especially important because aggressive fluid administration may aggravate pulmonary capillary leakage and worsen noncardiogenic pulmonary edema. Advanced supportive modalities, such as extracorporeal membrane oxygenation (ECMO), have shown survival benefits in selected patients with severe cardiopulmonary collapse refractory to conventional management [10].

Ribavirin is the most extensively studied antiviral agent for hantavirus infections. Clinical studies have demonstrated that early intravenous ribavirin administration may reduce mortality and disease severity in HFRS, particularly when initiated during the early febrile phase. However, evidence supporting its effectiveness in patients with HCPS remains inconclusive. Corticosteroids, immunomodulatory therapies, and passive immunotherapy have also been investigated, although their definitive clinical benefits have not yet been established. Recent therapeutic research has focused on monoclonal antibodies, antiviral compounds targeting viral replication pathways, and host-directed immunotherapeutic strategies [37,38].

Advances in molecular virology and immunology have significantly accelerated hantavirus vaccine research. Liu et al. [38] highlighted several emerging therapeutic and vaccine approaches, including DNA vaccines, recombinant vector vaccines, virus-like particle vaccines, and monoclonal antibody-based therapies. Experimental DNA vaccines targeting hantavirus glycoproteins have demonstrated promising immunogenicity and protective efficacy in animal models. Hooper et al. [37] demonstrated that a Sin Nombre virus DNA vaccine induced protective immune responses against both HCPS- and HFRS-associated hantaviruses, supporting the concept of a potential pan-hantavirus vaccine. Similarly, DNA vaccination with the Hantaan virus M gene elicited strong neutralizing antibody responses and cross-protection against multiple HFRS-associated hantavirus strains in preclinical studies [39].

Recent computational and molecular studies have further improved our understanding of viral pathogenesis and immune interactions. Noor et al. [40] identified conserved YXXΦ motifs and YXXΦ-like tetrapeptides among HFRS-causing hantaviruses, suggesting their potential roles in viral immune modulation and pathogenicity. These findings may contribute to the development of targeted antiviral agents and immunotherapeutic interventions.

Preventive strategies continue to be the cornerstone of hantavirus control. Public health measures primarily focus on minimizing human exposure to infected rodents and their contaminated environments. Recommended preventive practices include rodent control programs, environmental sanitation, safe food storage, proper disposal of rodent waste, and the use of personal protective equipment during high-risk occupational or environmental exposure. Public awareness campaigns, occupational education, and community-based surveillance programs are essential for reducing transmission risk [24].

Environmental monitoring and early outbreak detection systems are important components of disease prevention, particularly in endemic regions vulnerable to ecological and climatic changes. Because climate variability, deforestation, agricultural expansion, and urbanization significantly influence rodent population dynamics, integrated ecological surveillance may assist in predicting future outbreaks [24]. Continued interdisciplinary collaboration among clinicians, epidemiologists, virologists, and public health authorities is essential for advancing preventive strategies, improving therapeutic options, and reducing the global burden of hantavirus infections [41].

Implications, future recommendations, and limitations of the review

Hantavirus infection continues to represent a significant emerging zoonotic threat due to its high fatality rates, expanding geographical distribution, and diagnostic challenges. Improved surveillance, enhanced public health preparedness, and increased clinician awareness are essential for early recognition and effective management of outbreaks [1]. Advances in molecular diagnostics and immunopathogenesis research may contribute to the development of targeted antiviral therapies and effective vaccines in the future [37-39]. Further multicenter and longitudinal studies are recommended to better understand host immune responses, viral evolution, and regional epidemiological variations. Strengthening interdisciplinary collaboration among virologists, epidemiologists, clinicians, and public health authorities is also necessary for comprehensive disease control.

However, this review has certain limitations. As a narrative review, the possibility of selection bias cannot be completely excluded. Additionally, variations in study design, diagnostic methods, and regional reporting systems among the included studies may limit direct comparison of findings. Despite these limitations, the present review provides a comprehensive overview of the current perspectives on hantavirus infection and highlights important directions for future research and clinical practice.

## Conclusions

Hantavirus infection continues to represent an important public health concern with significant clinical and epidemiological implications. Advances in the understanding of disease biology and clinical characteristics have improved knowledge of the infection; however, challenges remain in its timely recognition and management. Ongoing research is focused on improving diagnostic approaches, therapeutic options, and preventive strategies. Strengthening surveillance, increasing awareness, and fostering collaborative research efforts are essential for enhancing disease control and reducing its impact. Continued progress in these areas may contribute to improved prevention, diagnosis, and management of hantavirus infection in the future.
